# Up-Regulation of RIP3 Alleviates Prostate Cancer Progression by Activation of RIP3/MLKL Signaling Pathway and Induction of Necroptosis

**DOI:** 10.3389/fonc.2020.01720

**Published:** 2020-08-26

**Authors:** Ke-jie Wang, Kai-yun Wang, Hui-zhi Zhang, Xiang-yu Meng, Jun-feng Chen, Ping Wang, Jun-hui Jiang, Qi Ma

**Affiliations:** ^1^Translational Research Laboratory for Urology, The Key Laboratory of Ningbo City, Ningbo First Hospital, The Affiliated Hospital of Ningbo University, Ningbo, China; ^2^Comprehensive Urogenital Cancer Center, Ningbo First Hospital, The Affiliated Hospital of Ningbo University, Ningbo, China; ^3^Medical School, Ningbo University, Ningbo, China; ^4^Department of Pathology, Ningbo Diagnostic Pathology Center, Ningbo, China; ^5^Department of Urology, Ningbo First Hospital, The Affiliated Hospital of Ningbo University, Ningbo, China

**Keywords:** cell death, necroptosis, RIP3, MLKL, prostate cancer

## Abstract

**Background:**

The receptor-interacting protein kinase 3 (RIP3/RIPK3) was recently found to be a critical regulator of programmed necrosis/necroptosis. However, the biological role and clinical significance of RIP3 in prostate cancer remain obscure.

**Methods:**

Western blotting and QRT-PCR were performed to detect the level of RIP3 in prostate cancer cells. Fixed cancer tissue and normal tissue specimens were subjected to immunohistochemical analysis of RIP3. Cell migration and invasion abilities were evaluated by transwell assays. *In vitro* proliferative ability was examed by MTS. And *in vivo* nude mice model were used to evaluate the effect of RIP3 ectopic expression on proliferative capability. Cell cycle of prostate cancer cells were analyzed by flow cytometry. Changes in some related proteins caused by RIP3 overexpression were explored using Western blotting.

**Results:**

RIP3 was significantly down-regulated in prostate cancer cell lines and clinical prostate tumor samples. And over-expressing RIP3 suppressed the migration and invasion of prostate cancer cells. Two important matrix metalloproteinases MMP2, MMP9 which enables the destruction of the histological barrier of tumor cell invasion and three mesenchymal markers Vimentin, fibronectin, and N-cadherin were under-expressed due to the overexpression of RIP3, but the E-cadherin level which is the epithelial marker was increased. Furthermore, our results also showed that RIP3 can inhibit the proliferation and tumorigenicity of prostate cancer cells both *in vitro* and *in vivo* by phosphorylating MLKL, which were reversed by MLKL inhibitor treatment, indicating that necroptosis was involved in cell death.

**Conclusion:**

Taken together, these findings indicated that RIP3 is responsible for the progression of prostate cancer, suggesting that RIP3 might have the potential to be a prognostic marker or a therapeutic target against prostate cancer.

## Background

Metastatic prostate cancer (mPCa) is one of the leading cancers responsible for male fatalities worldwide (1). In its early stages, androgen deprivation (ADT) is the mainstay therapy for mPCa, but with the progression of the prostate cancer, the majority of the patients will develop resistance to primary ADT, leading to an aggressive stage termed “metastatic castration-resistant prostate cancer”(mCRPC) (2). Although the molecular mechanism behind the progression of mPCa has been widely investigated, no effective method to control mCRPC currently exists. Further studies are still necessary to discover novel and effective therapeutic strategies.

Programmed cell death (PCD) is an important cellular mechanism whose dysregulation is involved in many kinds of human pathology and especially involved in the formation and progression of tumors. Induction of PCD through the activation of caspases (apoptosis) is the most researched death process in most cell types (3). In recent years, an alternative form of PCD termed “necroptosis” that operates without detectable caspase activity has been discovered (4, 5). In this process, the Receptor-interacting protein 3 (RIP3) is a crucial part of the cellular machinery that induces necroptosis. RIP3 is a Ser/Thr kinase that contains a homologous N-terminal kinase domain and a unique C-terminal domain (6, 7). Activation of the necroptosis involves the formation of a complex containing RIP3 and its family number, RIP1, as well as the recruitment and phosphorylation of mixed lineage kinase domain-like protein (MLKL), which triggers its oligomerization and plasma membrane localization, eventually leading to the rupture of the cell membrane and cell death (8–10).

It has been reported that the expression of RIP3 in tumor cells and tissues is often silenced due to genetic methylation in the RIP3 promoter. Furthermore, previous reports have also shown that various compounds such as shikonin (11), polyinosinic–polycytidylic acid (12), and resibufogenin (13) exert antitumor effects by inducing necroptosis. The susceptibility/resistance of tumor cells to necroptosis is primarily determined by the expression of the kinase RIP3, suggesting that RIP3 can be used in a novel strategy that targets the vulnerable points in cancer. Although RIP3 is known to have a key role in several cancers, the role of RIP3 in prostate cancer is still obscure.

In our present study, we showed that RIP3 was significantly down-regulated in human prostate cancer and negatively associated with cancer progression. Down-regulation of RIP3 was also associated with higher degrees of malignancy in prostate cancer cell lines. Over-expression of RIP3 markedly reduced the proliferation and tumorigenesis of prostate cancer cells *in vitro* and *in vivo*. Additionally, we found that the tumor’s ability to migration and invasion was significantly inhibited in RIP3-overexpressed cells. Finally, we demonstrated that cycle arrest and RIP3-induced necroptosis decreased the tumor’s proliferative capacity. Taken together, our study suggested that RIP3 has an essential role in inducing cell death and that RIP3 might be a potential target for the treatment of prostate cancer.

## Materials and Methods

### Tissue Samples

Prostate tumor and adjacent non-tumor tissue samples were obtained from the Department of Pathology, Ningbo Diagnostic Pathology Center. The study protocol was approved by the Ethical Committee of Ningbo First Hospital, and the information written consent was obtained from all the subjects.

Clinical data were obtained from medical records within Ningbo first Hospital. The clinicopathological characteristics of the included patients are summarized in Table 1.

**TABLE 1:** Association between the expression levels of RIP3 and clinic pathological features in prostate cancer.

Characteristic	Patients Number	RIP3 Expression Level		*P*
		Low (%)	High (%)	
Nomal vs Cancer				0.001***
Normal	21	3 (14.3)	18 (85.7)	
Cancer	84	45 (53.6)	39 (46.4)	
PSA Level				0.330
<10	9	5 (55.6)	4 (44.4)	
10–50	36	16 (44.4)	20 (55.6)	
>50	39	24 (61.5)	15 (38.5)	
Gleason				0.739
< = 7	35	18 (51.4)	17 (48.6)	
>7	49	27 (55.1)	22 (44.9)	
Tumor metastasis				0.01*
Metastasis	38	26 (68.4)	12 (31.6)	
Non-metastatic	45	18 (40.0)	27 (60.0)	
Unknown	1			
Biochemical recurrence				
Yes	40	25 (62.5)	15 (37.5)	0.118
No	44	20 (45.5)	24 (54.5)	

### Immunohistochemical Staining

The tissue was fixed with 4% paraformaldehyde, embedded in paraffin, and then cut into 5 μm samples. Immunohistochemical staining was performed using a classic manufacturer’s protocol. Briefly, the paraffin sections were dewaxed in xylene for 30 min and then hydrated by successively placing them in a series of concentration gradient ethanol aqueous solutions. The samples were heated in 10 mM citrate buffer (pH 6.0) for 15 min to complete antigen recovery. Next the endogenous peroxidase activity was blocked by incubation in 3% hydrogen peroxide for 10 min, and the sections were finally incubated overnight at 4°C in 1: 100 dilution of anti-RIP3 antibody (abcam, ab56164).

### Cell Culture

The prostate cancer cell lines LNCaP, PC3, 22RV1, and DU145 used in our study were from American Type Culture Collection (United States) and grown in F-12K (Gibco), DMEM (HyClone), or RPMI 1640 (HyClone) medium containing 10% heat-inactivated fetal bovine serum (ExCell Bio, Shanghai, China).

### Generation of Stable Cell Lines

Receptor-interacting protein 3-expressing lentivirus with GFP and puromycin resistance markers were purchased from Genechem company (Shanghai, China). The lentiviral vector system consists of three plasmids: GV492, pHelper 1.0, and pHelper 2.0 vectors. GV492’s manual can be downloaded at the following URL: http://www.genechem.com.cn/index/supports/zaiti_info.html?id=82.

Stable cell lines were established according to the manufacturer’s protocol. Briefly, PC3 and 22RV1 cells were transfected by lentiviruses vector production, and the cell culture medium was replaced with complete medium after 24 h. Lentiviruses transfected-PC3 and 22RV1 cells were selected by complete medium containing 4 μg/ml and 3 μg/ml puromycin after 72 h. RIP3 overexpression was confirmed by western blotting.

### Western Blot

Cells were lysed in RIPA buffer containing 1% protease inhibitor and 1% phosphatase inhibitor according to the manufacturer’s protocol. After centrifugation (12,000 rpm, 20 min), the supernatant was collected, and the protein concentration was detected by the BCA Protein Assay Kit. Proteins of different molecular weights were separated by SDS-PAGE gel electrophoresis, and then transferred to PVDF membrane After blocking with TBST containing 5% skim milk for 2 h, the membrane was incubated with the primary antibody at 4°C overnight, the secondary antibody was coupled with a suitable HRP.

The following primary antibodies were used: RIP3 (ab56164), β-actin (ab115777), MMP2 (ab37150), MMP9 (abab38898), Fibronectin (ab2413), Caspase 8 (ab25901), MLKL (ab183770), and p-MLKL (ab187091) were obtained from Abcam, Bax (#5023), Bcl-2 (#2870), Caspase 9 (#9502), GAPDH (#2118), Vimentin (#5741), Cyclin A2 (#4656), Cyclin B1 (#4135), CDK1/CDC2 (#9116), N-cadherin (#13116), and CDK2 (#2546) were obtained from CST. The dilution concentration of all primary antibodies were 1:1000. The secondary antibodies were coupled to horseradish peroxidase (Boster, Wuhan, China, working concentration 1: 5000) and detected by chemiluminescence.

### Quantitative Real-Time PCR

Total RNA was extracted from prostate cancer cells using Trizol reagent (Invitrogen, United States), followed by cDNA preparation using a reverse transcription kit (Thermo, United States). The cDNAs were amplified by qRT-PCR using SYBR Green PCR Master Mix (Roche, United States) on a LightCycler480 system, and relative abundance was determined using the ΔCt method.

The PCR primers were shown in Table 2.

**TABLE 2:** Oligonucleotide sequence of primer set used to amplify in each cDNA.

Gene	Forward primer sequence (5′-3′)	Reverse primer sequence (5′-3′)
*RIP3*	CTGAGTGGCTAAACAAACT GAATC	AGGTAGGGCTGGGCATCTG
β*-actin*	CACAGAGCCTCGCCTTTG	ATAGGAATCCTTCTGACCCATG

### Invasion and Migration Assays

Cellular invasion and migration were conducted using transwell chambers (8 μm pore diameter). Cells were seeded in a serum-free media (2 × 10^5^ cells/well) in the upper chambers with filters coated with BD Matrigel. The cells were added to the upper chamber and incubated at 37°C for 48 h. The surface cells were then wiped off with Q-tip, and the cells invaded through membrane were fixed with paraformaldehyde and stained with crystal violet. Crystal violet-stained infiltrated cells were then decolorized with glacial acetic acid, and the number of cells was indirectly quantified using the absorbance at 570 nm. The migration test was similar to the experimental method described above, with only minor modifications: (i) the seeding density was 1 × 10^5^ cells/well, and (ii) the Matrigel coating was omitted. Experiments were done in triplicate.

### Cell Proliferation Assay

Cell proliferation was detected by Cell Titer 96^®^ Aqueous One Solution Reagent (PROMEGA, Madison, United States) according to the manufacturer’s protocol. In brief, 3000 PC3, or 6000 22RV1 cells were plated in each well of a 96-well plate. Add 20 μl of Cell Titer 96^®^ Aqueous One Solution Reagent to each well after culturing for 4, 24, 48, 72, and 96 h. Absorbance was read at 490 nm after incubation for 3 h.

### Xenograft Model in Nude Mice

We subcutaneously injected 5 male BALB/c-nu mice (China Vital River Co.) with PC3 cells (2 × 10^6^ cells/100 μl) carrying the RIP3 expressing lentiviral vector, and injected the same number of PC3 cells with an empty vector into another 5 mice. The tumor volume was calculated with the following formula V = L × W^2^ × π/6 (V, volume; L, length; and W, width of tumor). On day 28 after implantation, the tumors were collected and weighed.

### Flow Cytometry Analysis of Cell Cycle

For the cell cycle analysis, the cells were collected and washed with PBS twice. Each sample was treated with a DNA staining solution containing 1 ml and 10 μl RNase A using the Cell Cycle Staining Kit (Multiscien-ces, Hang Zhou, China). The sample was then incubated for 30 min at 37°C in the dark and analyzed using flow cytometry.

### Necroptosis Assay by Lactate Dehydrogenase (LDH) Release

22RV1 (1 × 10^5^ cells/well), PC3 (5 × 10^4^ cells/well) prostate cancer cells were seeded onto a 96-well microplate and cultured for 24 h. The lactate dehydrogenase cytotoxicity assay kit (Beyotime Biotech, Nanjing, China) was used to detect cell mortality. The detection method was performed according to the instructions of the kit. The absorbance value of each sample was read at 490 nm.

### Statistical Analysis

ANOVA was used to evaluate the differences between groups. Two-sided Student’s *t*-test was used to compare differences between two groups. Kaplan-Meier analysis was used to plot survival curves. Differences were considered significant if *p* < 0.05. ^∗^*p* < 0.05, ^∗∗^*p* < 0.01, and ^∗∗∗^*p* < 0.001.

## Results

### RIP3 Is Decreased in Prostate Cancer, and the Low RIP3 Level Is a Risk Factor for the Prognosis of Prostate Patients

**FIGURE 1:**
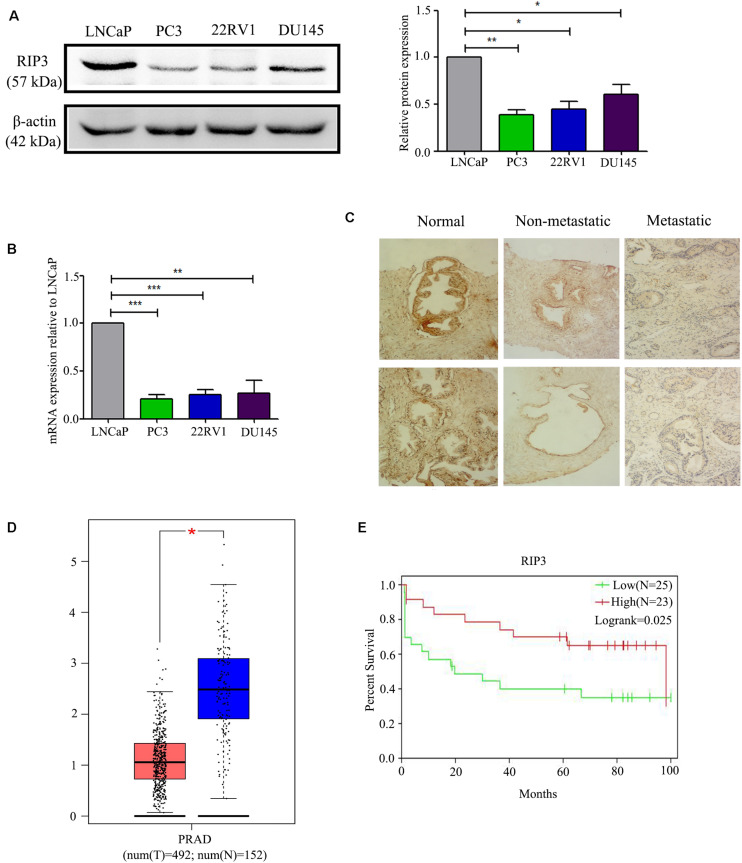
RIP3 level is frequently down-regulated in human prostate cancer. **(A,B)** Western blotting and QRT-PCR were performed to detect the level of RIP3 in prostate cancer cells (LNCaP, PC3,22RV, and DU145). Data shows average of 3 independent experiments ± SEM. **(C)** Fixed cancer tissue and normal tissue specimens were subjected to immunohistochemical analysis of RIP3. Higher expression of RIP3 in paraffin-embedded normal tissue than tumor tissue. Representative images were captured at a magnification of 100x. **(D)** The expression of RIP3 was analyzed in 492 prostate tumor tissues and 152 normal samples by using TCGA and GTEx database. **(E)** The overall survival curves of prostate cancer patients, data from GEO database (GSE70769).

To detect the level of RIP3 in prostate cancer, Western Blotting was performed in different prostate cell lines. Compared to LNCaP cells with low malignancy, the cell lines PC3, 22RV1, DU145 with higher malignancy were found to have down-regulated RIP3 (Figure 1A). To further verify this, we used the QRT-PCR to detect the expression of RIP3 in these cancer cells, and the results were very similar to the protein levels (Figure 1B). To explore the clinically significant difference of RIP3 in prostate cancer, pathology samples were analyzed using immunohistochemistry with a specific RIP3 antibody (Figure 1C). Based on the staining results, RIP3 had higher levels in normal tissues than cancer tissues (*P* = 0.001; Table 1). Meanwhile, We discovered the same result by analyzing the TCGA data set (Figure 1D). Surprisingly, we found that RIP3 expression was statistically significantly associated with tumor metastasis (*P* = 0.01; Table 1). In non- mPCa, the positive rate of RIP3 was significantly higher than that of mPCa (60.0% vs. 31.6%). Furthermore, patients with low RIP3 expression had a poorer survival (Figure 1E). Taken together, these suggested that RIP3 may be an important tumor suppressor and that its expression may be closely related to tumor growth and metastasis.

### Overexpression of RIP3 Inhibits the Migration and Invasion of Prostate Cancer Cells

**FIGURE 2:**
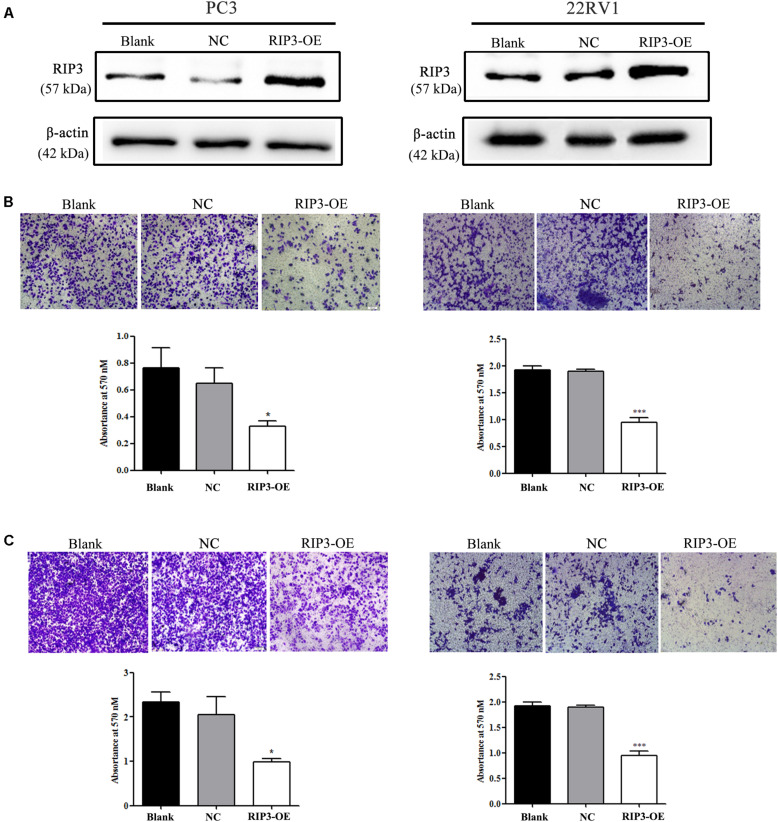
Overexpression of RIP3 inhibits migration and invasion of prostate cancer cells. **(A)** Western blotting was performed to exam the expression of RIP3. Lentiviral vector transfected with RIP3 significantly increased the expression of RIP3 in PC3 and 22RV1 cells. **(B)** Cell migration was evaluated by transwell assay, the number of migrated cells was obviously decresed in RIP3 transfected groups. **(C)** Cell invasion ability was evaluated by matrigel transwell assay, the number of invaded cells was also drastically reduced in RIP3 overexpession groups. Representative images were captured at a magnification of 40x. All tests were repeated three times. Error bars represent the SEM.

To elucidate the biological functions of RIP3 in prostate cancer, we established a lentivirus vector-mediated RIP3 over-expressed (RIP3-OE) stable prostate cancer cell lines using PC3 and 22RV1 cells. Western Blotting was then performed, and we found that compared to the control groups, the expression of RIP3 in lentivirus-infected groups were significantly higher (Figure 2A). Next, the effect of RIP3 overexpression on cell migration and invasion in prostate cancer cells was determined using the transwell assay. As shown in Figure 2B, the number of migrated cells was significantly decreased in RIP3-OE cells. Similar results could be seen in Figure 2C, the number of invading cells was also drastically reduced in RIP3-OE groups. These results suggested that overexpression of RIP3 could inhibit the migration and invasion of prostate cancer cells.

### RIP3 Functions as a Tumor Suppressor to Inhibit Prostate Cancer Cell Proliferation

**FIGURE 3:**
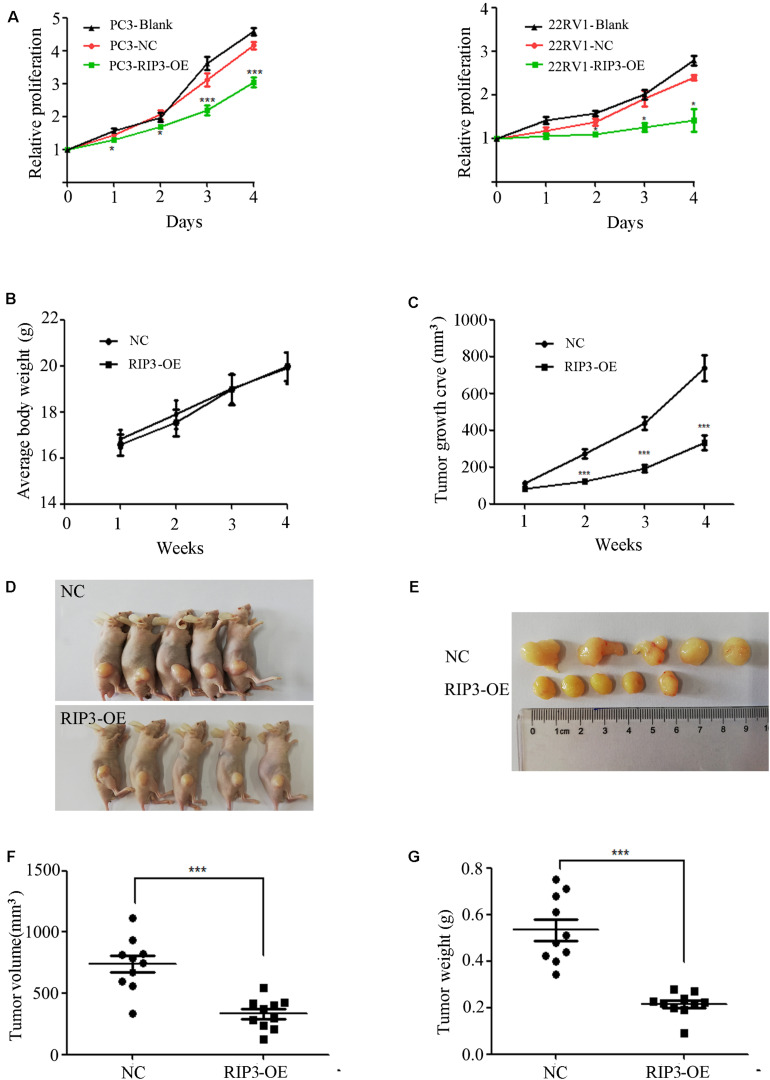
Overexpression of RIP3 suppresses prostate cancer cells growth *in vitro* and *in vivo*. **(A)** MTS assay was performed to exam the proliferative ability. Upregulated RIP3 caused tumor cells to present with lower proliferative abilities than the negative control and blank control in PC3 and 22RV1 cells. Data shows average of 3 independent experiments ± SEM. Nude mice were injected with lentiviral-delivered RIP3 and control plasmid (NC)-infected PC3 cells. The weight of mice was showed in **(B)**. The tumor growth was measured intermittently and were depicted in the line chart **(C)**. On day 28 after injection of transfected PC3 cells, the tumors were collected for imaging **(D,E)**. Final volume and weight of the tumor were showed in **(F,G)**. The *in vivo* data was repeated twice, each time with 5 animals per group. All the values represent the means ± SEM.

To further determine the role of RIP3 in the regulation of prostate cancer progression, we evaluated the proliferation rate using MTS assay *in vitro*. We found that up-regulation of RIP3 reduced the tumor’s proliferative abilities more than the negative control and blank control (Figure 3A). Additionally, the effect of RIP3 on tumor xenograft growth in nude mice was investigated. As shown in the xenografted mice model (Figures 3B–G), mice were sacrificed at 4 weeks, and tumors were harvested, measured and weighed, the RIP3-OE groups showed significantly decreased tumor growth as compared with control xenografts. These results indicated that overexpression of RIP3 inhibited prostate cancer formation and growth.

### Overexpression of RIP3 Alters Migration and Invasion-Associated Proteins

**FIGURE 4:**
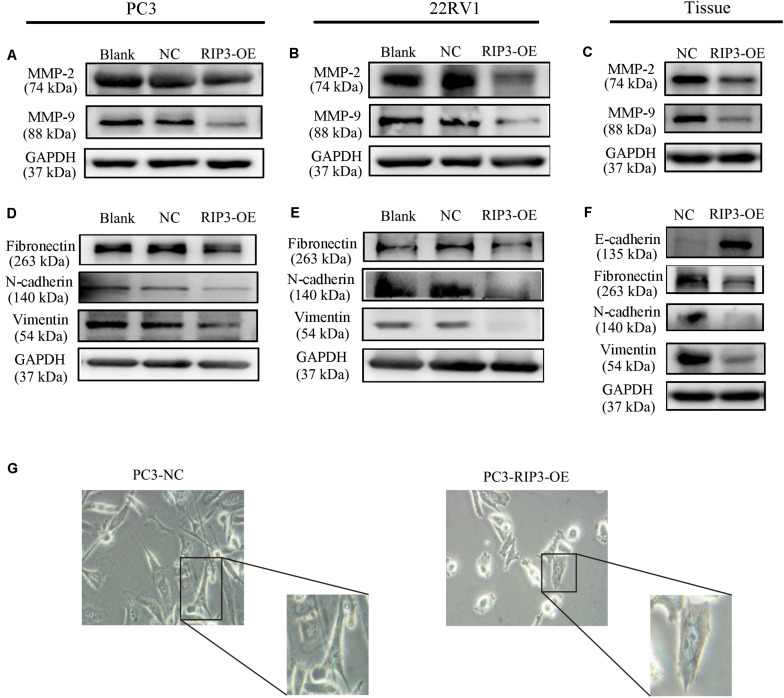
Overexpression of RIP3 alters migration and invasion-associated proteins. **(A–C)** MMP2 and MMP9 proteins were examed by Western blotting in PC3, 22RV1 cells and mouse tumor tissues. **(D–F)** The expression of proteins which related to epithelial-mesenchymal transition were determined by Western blot in PC3, 22RV1 cells, and mouse tumor tissues. The protein tests above were all repeated three times. **(G)** Morphology was analyzed by microscopy. Representative images were captured at a magnification of 200x.

MMPs play a key role in tumor invasion and metastasis as they can destroy the histological barrier against tumor cell invasion through the degradation of various protein components in the ECM (14). Among the various MMP family members, the gelatin-degrading enzymes MMP-2 and MMP-9 are considered the tumor-invasive components as their expression levels are often up-regulated in advanced stages of cancer (15). From the above results, we have observed that overexpression of RIP3 can inhibit the migration and invasion of prostate cancer cells, so we examined the expression levels of MMP2 and MMP9 in PC3, 22RV1 cells, and mouse tumor tissues, respectively. Unsurprisingly, we found that the expression levels of the two MMP proteins were reduced in the RIP3-OE groups (Figures 4A–C).

Tumor migration and invasion are often accompanied by epithelial-mesenchymal transition (EMT) which is a process that causes the epithelial cells to lose its epithelial characteristics and gain mesenchymal features (16). To assess how EMT-related proteins function during metastasis when RIP3 is overexpressed, we postulated that Vimentin expression levels like Fibronectin and N-cadherin, which are mesenchymal markers should be down-regulated during EMT. Just as we expected, our results showed that the expression of these mesenchymal markers decreased in RIP3-OE cells (Figures 4D,E). Furthermore, we checked these mesenchymal markers in the mouse tumor tissues samples and found that their expression levels were significantly decreased too. We also checked E-cadherin levels in the mouse tissue samples, which is an epithelial marker and found that the expression levels of E-cadherin were dramatically increased (Figure 4F). At the same time, we observed under the microscope that after high expression of RIP3, the tumor cells gradually lost the characteristics of mesenchymal cells, and changed from long, polygonal to spindle appearances (Figure 4G). Taken together, these data suggested that overexpression of RIP3 inhibits EMT both *in vitro* and *in vivo*.

### Overexpression of RIP3 Induces Cell Cycle G2 Arrest

**FIGURE 5:**
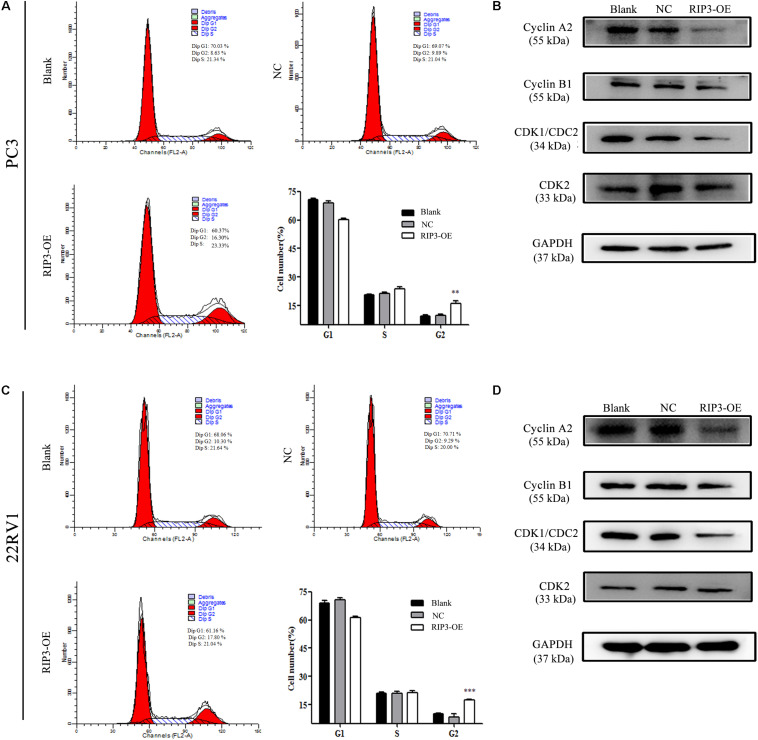
Overexpression of RIP3 induces cell cycle G2 arrest. **(A,C)** Cell cycle of PC3 and 22RV1 cells were analyzed by flow cytometry. RIP3 transfected groups showed a remarkable increase at the G2 phase compared with blank and NC groups. **(B,D)** Expression levels of cyclin A2, cyclin B1, CDK1/CDC2, and CDK2 in PC3 and 22RV1 cells were determined by Western blotting, using GAPDH as a control. All tests were repeated three times. Data shows average of 3 independent experiments ± SEM.

The cell cycle distribution was analyzed to investigate whether the inhibitory effects observed on the cell proliferation of PC3 and 22RV1 cells during upregulation of RIP3 was mediated by cell cycle alteration. The data showed that in comparison with the blank control group, the cell population was significantly increased at the G2 phase when RIP3 was upregulated (Figures 5A–C), indicating that overexpression of RIP3 arrests the cell cycle in G2 in PC3 and 22RV1 cells. We then detected several cell cycle-related proteins expression by Western blotting. The results showed that in PC3 and 22RV1 cells, up-regulated RIP3 reduced the expression of cyclin A_2_, cyclin B_1_, and CDK1/CDC2, which regulate the conversion of G2 to M phage (Figures 5B–D). However, the expression of CDK2, a protein that regulates the conversion from S to G2, did not change. These data suggested that overexpression of RIP3 induces G2 arrest by down-regulating the G2 to M transformed related proteins, then leading to cell proliferation inhibition.

### RIP3-Mediated Prostate Cancer Proliferation Inhibition Is via Necroptosis but Not via Apoptosis

**FIGURE 6:**
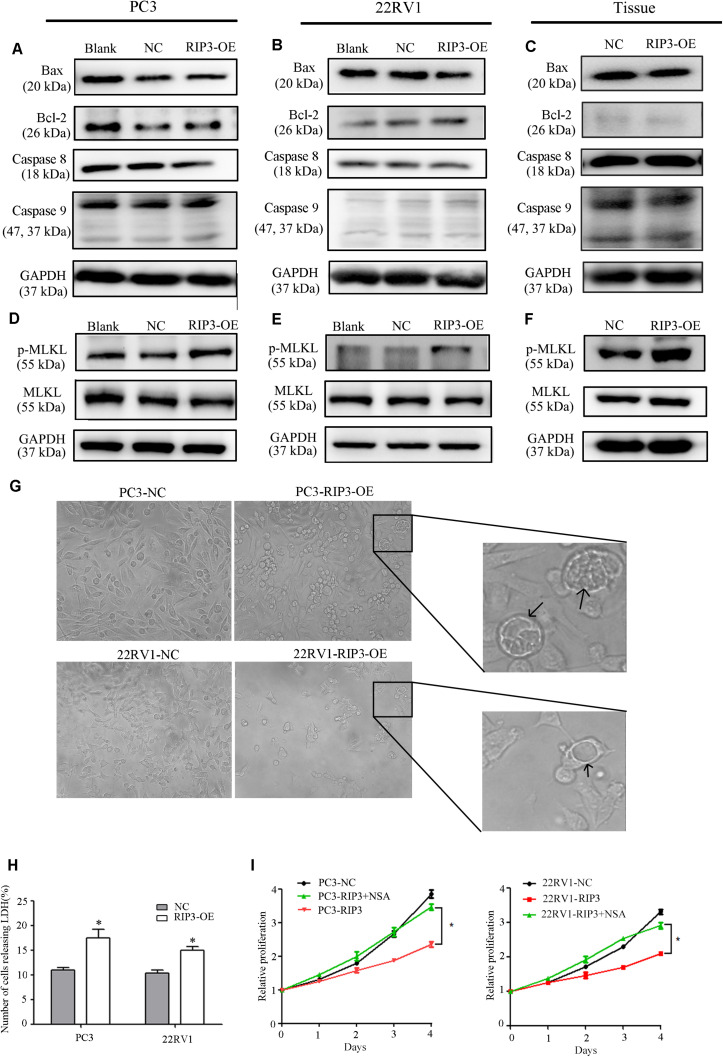
RIP3-mediated prostate cancer proliferation inhibition is via necroptosis but not via apoptosis. **(A–F)** Expression levels of Bax, Bcl-2, Caspase8, Caspase 9,MLKL, and p-MLKL in PC3, 22RV1 cells and mouse tumor tissues were determined by Western blotting, using GAPDH as a control. **(G)** Morphology was analyzed by microscopy. Arrowheads show cells with cytoplasmic swelling. Representative images were captured at a magnification of 80x **(H)** LDH release assay proved that the number of cell death in high-expression RIP3 groups were more than negative control. **(I)** PC3-RIP3 and 22RV1-RIP3 were treated with 5 μM NSA when the cells were seeded in 96-well plates, proliferation inhibition caused by high expression of RIP3 disappeared. Data are mean ± SEM of three replicates.

To further investigate how overexpressed RIP3 causes proliferation inhibition, we examined the endogenous apoptosis-related proteins Caspase 9, Bax, Bcl-2 and the exogenous apoptosis-related protein Caspase 8. We found no significant changes in the levels of these proteins in the RIP3-OE groups (Figures 6A–C). These results indicated that RIP3 did not inhibit the prostate cancer proliferation through apoptosis.

Unlike apoptosis, necroptosis is a type of caspase-independent PCD with morphological similarities to necrosis, and RIP3 is a key in this death process (17). It can phosphorylate its downstream necroptosis execute protein MLKL which eventually led to cell death (18–20). Therefore, we examined the total MLKL and p-MLKL levels in the PC3, 22RV1 and mouse tumor tissues samples, respectively. We found that there was no change in the total MLKL level in the RIP3-OE groups, but the p-MLKL level was significantly elevated compared with blank and negative controls (Figures 6D,E). Additionally, we analyzed the cell morphology by microscopy. The arrowheads indicate cytoplasmic swelling, which is an important feature of necroptosis (Figure 6G). Compared with apoptotic cell death, necroptosis does not cause condensed chromatins, shrinkage of the cell body or genome fragmentation, but its key feature is the deterioration of the cytoplasm membrane, which can be confirmed by LDH leakage (21, 22). Then we detected LDH release, and the result revealed that there was significantly increased cell death in the high-expression RIP3 groups than the negative control (Figure 6H). These data suggested that overexpression of RIP3 lead to prostate cancer cell death through the necroptosis pathway.

To further demonstrate this, we used the MLKL inhibitor Necrosulfonamide (NSA) to inhibit necroptosis and observed that overexpressed RIP3 no longer inhibited the tumor cells’ ability to proliferate (Figure 6I). In summary, the results suggest that RIP3-mediated prostate cancer proliferation inhibition is via necroptosis but not via apoptosis.

## Discussion

Necroptosis has been involved in various physiological and pathological processes, including inflammatory disease (23), ischemic injury (24), and neurodegeneration (25). RIP3 is a key molecule in the regulation of necroptosis. Interestingly, most cancer cells show a RIP3 defect, which enables them to inhibit necroptosis (26, 27). In the current study, we found that RIP3 was significantly down-regulated in prostate cancer cells with a higher degree of malignancy either at the protein level or the mRNA level (Figures 1A,B). Then, we collected 21 normal prostate biopsy specimens and 84 prostate cancer biopsy specimens and found that RIP3 expression was significantly reduced in prostate cancer compared with normal prostate biopsy specimens (Figure 1C). Surprisingly, we found that RIP3 expression is negatively correlated with tumor metastasis (*P* = 0.01; Table 1). In non- mPCa, the RIP3 expression levels were significantly higher than that of mPCa (60.0% vs. 31.6%).

At the same time, through TCGA and GEO databases, we also found that prostate cancer tissues have lower expression of RIP3 than normal tissues (Figure 1D). And patients with low RIP3 expression had a poorer survival (Figure 1E). These results were of great interest to us and so we continued to analyze the role of RIP3 in prostate cancer.

According to our clinical data, we first found that RIP3 dramatically reduced the tumor cells ability to invade and migrate (Figures 2B,C), and to understand the underlying mechanisms of RIP3 involvement in cell invasion and migration, we investigated the expression levels of the associated proteins. Degradation of the ECM and components of the basement membrane are initiated by MMPs, which play a critical role in tumor invasion and metastasis (15). Among the MMP family members, the gelatin-degrading enzymes MMP-2 and MMP-9 are associated with tumor ability to invade as their expression levels are often upregulated in the advanced stages of cancer (28). Our results showed that overexpression RIP3 led to significantly decreased MMP-2 and MMP-9 levels, which inhibited the progression of the tumor (Figures 4A–C).

Another typical feature of tumor migration and invasion is the EMT process, in which epithelial cells lose their epithelial characteristics and gain mesenchymal features (16, 29, 30). In this process, cells downregulate the expression of various epithelial cellular adhesion molecules like E-cadherin and gain mesenchymal properties by upregulating the expression of mesenchymal markers such as vimentin, fibronection and N-cadherin (31). Our results showed that overexpression RIP3 increased the expression of E-cadherin, and decreased expression of fibronectin, N-cadherin and vimentin to inhibition the EMT process (Figures 4D–F). We also observed morphological changes consistent with the alterations in the protein that high expression of RIP3 lead to the tumor cells gradually lost the characteristics of mesenchymal cells, and change from long, polygonal to spindle appearances (Figure 4G). Taken together, these protein changes further indicated that RIP3 can inhibit prostate cancer migration and invasion. However, how RIP3 regulates the expression of these proteins requires further study.

To further determine the role of RIP3 in the regulation of prostate cancer progression, cell proliferation was evaluated *in vitro* and *in vivo*. Experimental data showed that the tumor proliferation rate was significantly reduced in cells transfected with RIP3 and significantly inhibited tumor formation in the xenograft model (Figure 3). Next, we examined the cell cycle and related cyclins and found that cells were arrested in G2 phase, and the expressions levels of cyclin A2, cyclin B1, and CDK1/CDC2, which were related to the transition from the G2 phase to M phase, was dramatically decreased (Figure 5). Therefore, the results suggested that inhibition of tumor proliferation induced by RIP3 may be associated with cell cycle arrest.

Eliminating unwanted or damaged cells through cell death is essential for fundamental biological processes such as animal development, tissue homeostasis, and stress response. Deregulated cell death contributes to several human diseases including cancer. Apoptosis, which is the reverse process of cell proliferation, is one of the most common routes of cell death (32). Its occurrence depends on the activation of caspases and the bcl-2 family (33). However, we did not detect significant changes in the expression levels of bax, bcl-2, caspase8, and caspase9 proteins (Figures 6A–C), suggesting that RIP3-induced tumor proliferation inhibition is not associated with apoptosis. Recent studies demonstrated that PCD is not only confined to caspase-dependent apoptosis, but also includes necroptosis, a form of necrotic death governed by receptor-interacting protein 1 (RIP1), RIP3, and MLKL (34). RIP3 was shown to be a critical regulator of necroptosis (17) and is bound to RIP1 to form a necrosome that auto-phosphorylates and is subsequently activated, allowing itself to recruits and phosphorylates MLKL. This results in the oligomerization of MLKL and insertion of MLKL oligomers into the membrane, which disrupts the integrity of the plasma and intracellular membrane, leading to necroptotic death (18). In this process, the MLKL is the key player in the execution of cell death. Therefore, by analyzing the phosphorylation level of MLKL, we observed that the phosphorylation level of MLKL was significantly elevated when the cells were transfected with RIP3 (Figures 6D–F). When prostate cancer cells were treated with the MLKL inhibitor NSA, the inhibition of proliferation induced by overexpression of RIP3 stopped (Figure 6I), indicating that necroptosis is the main cause of tumor proliferation inhibition. Additionally, RIP3-OE cells exhibited several necrosis-like phenotype changes such as vacuoles which have distinctive characteristics that differentiate them from other types of cell death-like apoptosis (Figure 6G) (35). Similarly, LDH leakage, one of the indicators of necroptosis was significantly increased in the RIP3-OE group (Figure 6H). In summary, these results indicate that RIP3-mediated prostate cancer proliferation inhibition is via necroptosis but not via apoptosis.

## Conclusion

**FIGURE 7:**
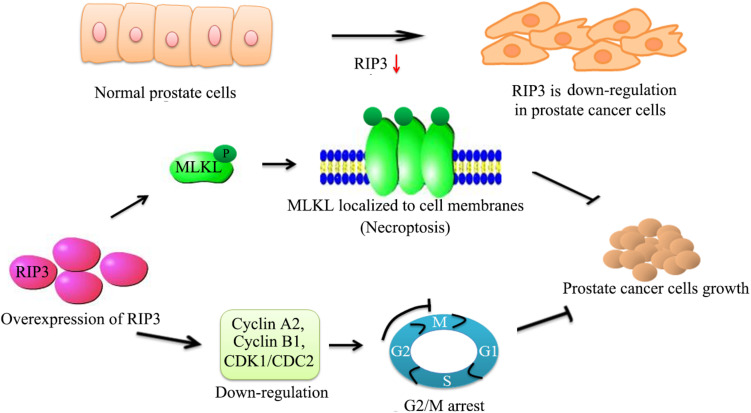
Schematic representation of alleviation prostate cancer progression via overexpresion of RIP3. We have initially confirmed that RIP3 plays an important tumor suppressive role in prostate cancer. Overexpression of RIP3 increases phosphorylation of MLKL, which resulting in oligomerization of MLKL. Then MLKL oligomers insert into cell membranes to disrupt the intracellular membrane integrity to lead necroptotic death. Furthermore, RIP3 reduces the expression of cyclin A2, cyclin B1, and CDK1/CDC2, which regulate the conversion of G2 to M phage to lead cell cycle arrest. Thus, RIP3 controls the progression of prostate cancer by blocking cell cycle and promoting necroptosis.

To our knowledge, our study demonstrated that RIP3 has a key role in prostate cancer progression for the first time. RIP3 inhibits the migration, invasion, and proliferation of prostate cancer cells significantly. Furthermore, RIP3 suppresses tumors through cell arrest and activation of the RIP3/MLKL signaling pathway to inducing necroptosis (Figure 7). Our findings indicate that RIP3 is a potential prognostic marker or a therapeutic target in the fight against prostate cancer.

## Data Availability Statement

All datasets generated for this study are included in the article/Supplementary Material.

## Ethics Statement

The studies involving human participants were reviewed and approved by the Ethics Committee of Ningbo First Hospital. The patients/participants provided their written informed consent to participate in this study. The animal study was reviewed and approved by Committee of Ningbo University. Written informed consent was obtained from the individual(s) for the publication of any potentially identifiable images or data included in this article.

## Author Contributions

QM, JJ, PW, and K-JW were responsible for the experimental design. K-JW, JC, and XM contributed to the execution of experiments and manuscript composition. QM, K-YW, and HZ participated in performing the data statistics. All authors have contributed to and approved the final manuscript.

## Conflict of Interest

The authors declare that the research was conducted in the absence of any commercial or financial relationships that could be construed as a potential conflict of interest.

## Supplementary Material

FIGURE S1 Changes in cell migration, invasion and cell cycle arrest have nothing to do with cell death, but are related to RIP3 expression. The PC3 cells of the experimental group and the control group were first cultured with NSA (5 μM) for 24 h. **(A)** Cell migration was evaluated by transwell assay (NSA was not removed in the process). **(B)** Cell invasion ability was evaluated by matrigel transwell assay (NSA was not removed in the process). Representative images were captured at a magnification of 40x. **(C)** Cell cycle of PC3 cells were analyzed by flow cytometry after treating with NSA for 24 h.

FIGURE S2 Results of the first *in vivo* experiment.

FIGURE S3 Results of the second *in vivo* experiment. Nude mice were injected with lentiviral-delivered RIP3 and control plasmid (NC)-infected PC3 cells. The weight of mice was showed in **(A)**. The tumor growth was measured intermittently and were depicted in the line chart **(B)**. On day 28 after injection of transfected PC3 cells, the tumors were collected for imaging **(C,D)**. Final volume and weight of the tumor were showed in **(E,F)**. The values represent the means ± SEM.
