# Supplementary figures and images for: Up-Regulation of RIP3 Alleviates Prostate Cancer Progression by Activation of RIP3/MLKL Signaling Pathway and Induction of Necroptosis
Source: Front Oncol. 2020 Aug 26;10:1720. doi: 10.3389/fonc.2020.01720 (PMC7480187; doi:10.3389/fonc.2020.01720)

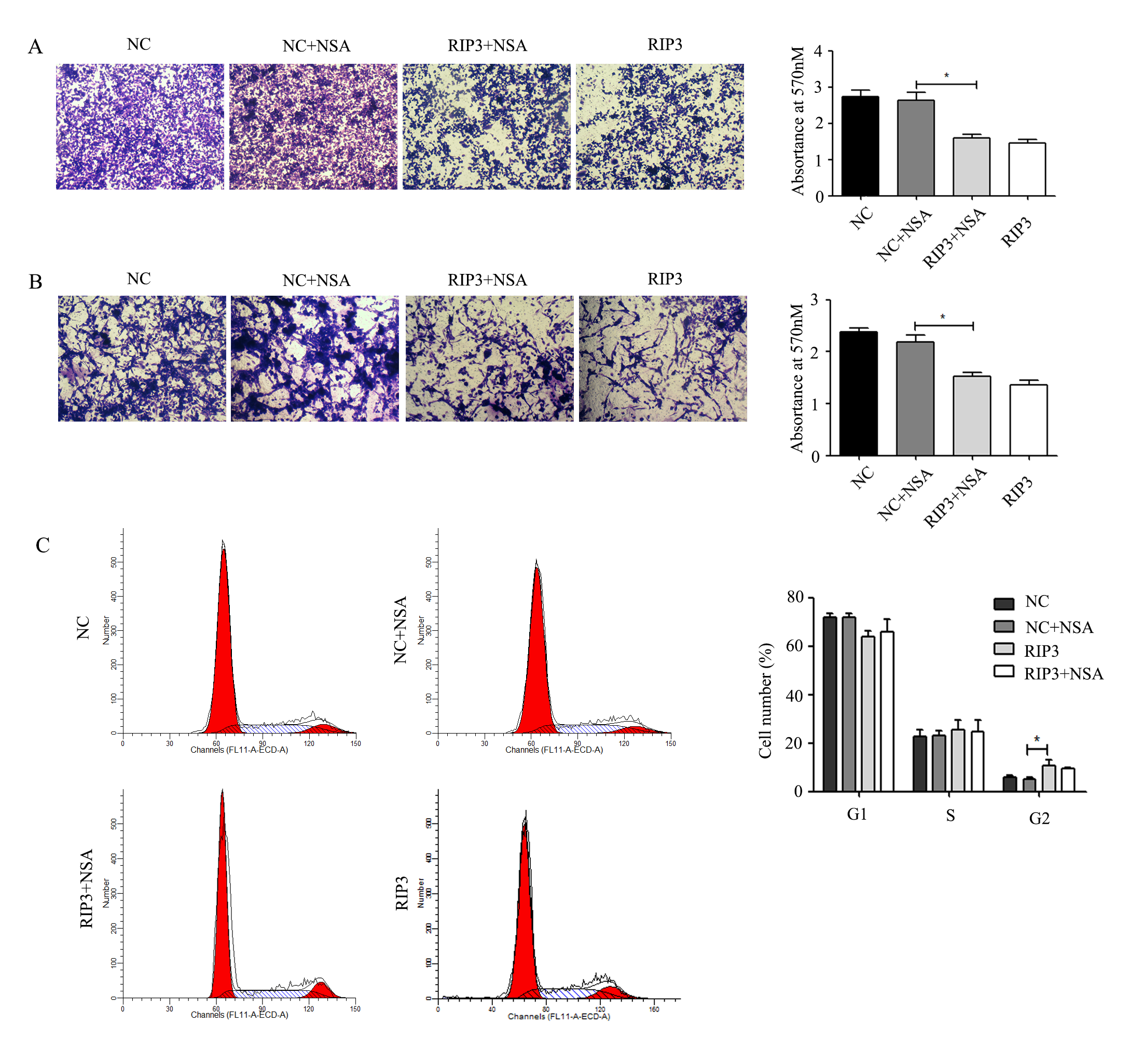

Supplement: FIGURE S1 — Changes in cell migration, invasion and cell cycle arrest have nothing to do with cell death, but are related to RIP3 expression. The PC3 cells of the experimental group and the control group were first cultured with NSA (5 μM) for 24 h. (A) Cell migration was evaluated by transwell assay (NSA was not removed in the process). (B) Cell invasion ability was evaluated by matrigel transwell assay (NSA was not removed in the process). Representative images were captured at a magnification of 40x. (C) Cell cycle of PC3 cells were analyzed by flow cytometry after treating with NSA for 24 h. [file Image_1.TIF]

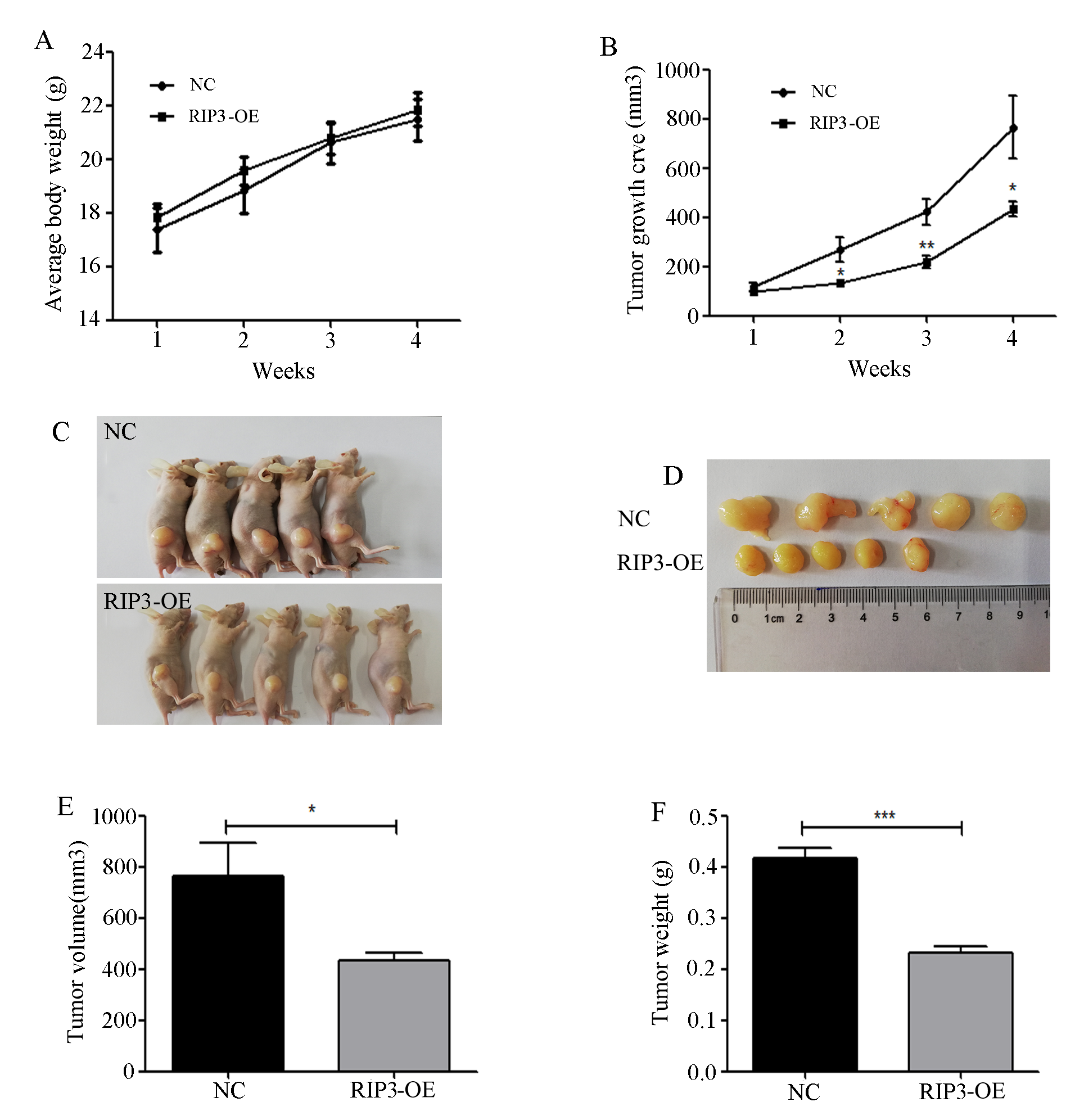

Supplement: FIGURE S2 — Results of the first in vivo experiment. [file Image_2.TIF]

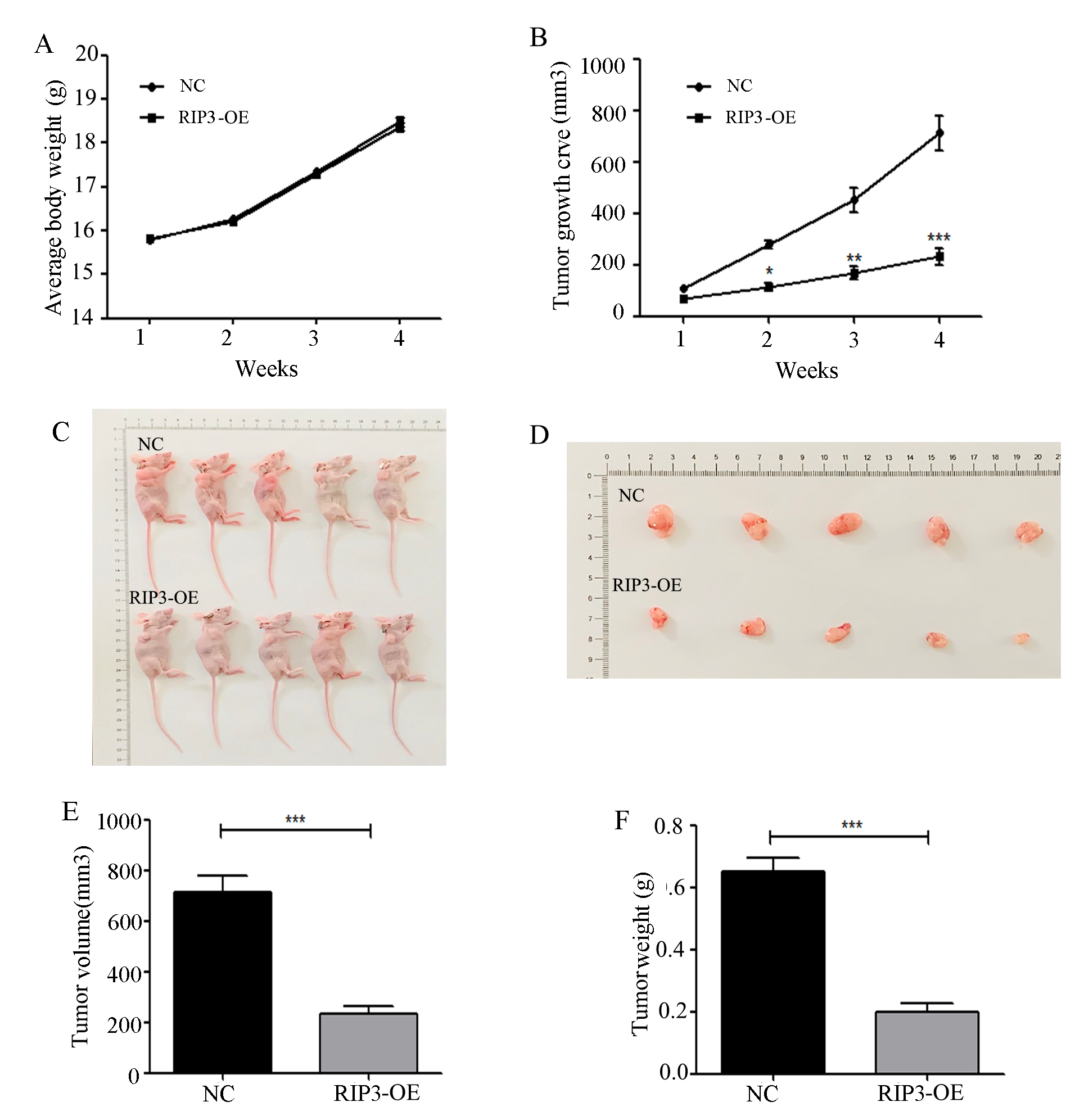

Supplement: FIGURE S3 — Results of the second in vivo experiment. Nude mice were injected with lentiviral-delivered RIP3 and control plasmid (NC)-infected PC3 cells. The weight of mice was showed in (A). The tumor growth was measured intermittently and were depicted in the line chart (B). On day 28 after injection of transfected PC3 cells, the tumors were collected for imaging (C,D). Final volume and weight of the tumor were showed in (E,F). The values represent the means ± SEM. [file Image_3.TIF]
